# Superior cerebellar artery occlusion remaining after thrombectomy for acute basilar artery occlusion

**DOI:** 10.1038/s41598-023-50023-5

**Published:** 2023-12-16

**Authors:** Byung Hyun Baek, Yun Young Lee, Seul Kee Kim, Woong Yoon

**Affiliations:** Department of Radiology, Chonnam National University Medical School, Chonnam National University Hospital, 42 Jebong-ro, Dong-gu, Gwangju, 61469 Republic of Korea

**Keywords:** Diseases, Neurology

## Abstract

To investigate the incidence and impact of superior cerebellar artery (SCA) occlusion remaining after thrombectomy for acute basilar artery occlusion (BAO). We retrospectively analyzed data from 116 patients who underwent thrombectomy for BAO. The patency of SCA was assessed on final angiograms. Clinical and radiologic data of the patients were retrieved from a prospectively collected database and analyzed. All patients underwent pretreatment and follow-up DWI to detect new infarctions in SCA territory. Ten patients (8.6%) had SCA occlusions on final angiograms. Of these, two patients had bilateral occlusions. A new infarction with a diameter ranged from 4 to 11 mm in corresponding SCA territory occurred in 5 of 10 patients. No patients with SCA occlusions experienced symptomatic cerebellar hemorrhage or malignant cerebellar infarction. Nine of 12 SCA occlusions showed spontaneous recanalization on follow-up CT angiography. Four of 10 patients showed 90-day favorable outcome (mRS 0–3) and 90-day mortality occurred in one patient. SCA occlusions remaining after thrombectomy for acute BAO had a benign clinical course. Most of these lesions recanalized spontaneously. Our study suggests that attempts to recanalize remnant SCA occlusion may be unnecessary after basilar artery thrombectomy.

## Introduction

Since two recent randomized trials demonstrated the superiority of endovascular treatment over medical treatment for treating patients with an acute basilar artery occlusion (BAO), it is now expected that endovascular thrombectomy will be increasingly used to treat these patients^1,2^. When performing thrombectomy for BAO, little attention has been paid to the patency of cerebellar arteries and the clinical impact of their occlusion. Determining the clinical significance of an occlusion of each cerebellar artery may help prognostication after thrombectomy in posterior circulation stroke. To date, however, there have been no reports regarding the clinical consequences of occlusion of cerebellar arteries that remains after thrombectomy for BAO.

The superior cerebellar artery (SCA) is one of the major posterior fossa arteries and supplies the whole superior aspect of the cerebellar hemisphere, the superior vermis, the largest part of the cerebellar deep white matter, and the lateral and posterior regions of the midbrain^3,4^. The SCA is the most consistent cerebellar artery, and thus no visualization of the SCA confidently indicates its occlusion^5,6^. SCA occlusions may be observed on final angiograms after successful removal of clots from the basilar artery, especially when clot lodged in the distal segment of the basilar artery. Under these circumstances, neurointerventionalists need to decide whether to perform additional interventional procedure to recanalize the remnant SCA occlusions or not. However, the optimal management of remained SCA occlusions has not yet been reported. Thus, this study aimed to investigate the incidence and clinical impact of remaining SCA occlusion after endovascular thrombectomy for acute stroke due to BAO.

## Materials and methods

### Patients

Between January 2011 and December 2020, a total of 128 consecutive patients with acute BAO received endovascular thrombectomy at a comprehensive stroke center. Of these, 5 patients without pretreatment diffusion-weighted imaging (DWI), 6 patients without posttreatment DWI, and one patient without 90-day modified Rankin Scale (mRS) data were excluded from the study. Clinical and radiologic data of the remaining 116 patients were retrieved from a prospectively collected database and analyzed. The institutional review board (Chonnam National University Hospital IRB, IRB number CNUH-2022-412) approved this study and waived the requirement to obtain informed consent on the basis of the retrospective study design. This study was performed in line with the principles of the Declaration of Helsinki. All procedures and study methods were carried out in accordance with relevant guidelines and regulations.

### Endovascular therapy

Inclusion criteria for endovascular thrombectomy were as follows: occlusion of the basilar artery confirmed on catheter angiography, presentation within 12 h of stroke onset or last-seen-well, baseline National Institutes of Health Stroke Scale (NIHSS) score ≥ 4, and no intracranial hemorrhage on pretreatment CT. Intravenous thrombolysis with recombinant tissue plasminogen activator was performed in eligible patients before thrombectomy. Endovascular procedures were performed under local anesthesia in most patients. Conscious sedation was used at the discretion of the neurointerventionalists. Endovascular thrombectomy was performed with a stent retriever, a large-bore aspiration catheter, or both (e.g., simultaneous use of a stent retriever and an aspiration catheter). Intracranial balloon angioplasty and/or stenting was performed to treat underlying severe (≥ 70%) atherosclerotic stenosis, if needed. We did not perform additional recanalization procedures for SCA occlusion even if it was revealed on final angiograms. All patients underwent nonenhanced CT scans immediately after and at 24–72 h after endovascular therapy.

### Imaging analysis

All patients included in the study underwent pretreatment DWI and follow-up DWI within 3 days after the endovascular procedure. DWI examination were performed with a 1.5 T system (Signa HDxt; GE Medical Systems, Milwaukee, Wisconsin, USA) or a 3.0 T system (Ingenia 3.0 T CX, Philips Medical Systems, Best, Netherlands). At DWI, the SCA territory infarction was defined as diffusion-restricted lesions in the upper half of the ipsilateral cerebellar hemisphere or middle cerebellar peduncle or in the lateral or posterior region of the midbrain^4,7^. The presence or absence and types of SCA territorial infarction (cerebellar or midbrain) were recorded in each patient. We also assessed the posterior circulation Alberta Stroke Program Early CT Score (pc-ASPECTS) on pretreatment DWI.

The site of BAO was classified as proximal, middle, or distal on initial angiograms in accord with previous studies^8^. Overall reperfusion status was assessed on final angiograms according to the modified Thrombolysis In Cerebral Ischemia (TICI) grade. Successful reperfusion was defined as an modified TICI score of 2b or 3. The patency or occlusion of the superior cerebellar arteries was assessed on final angiograms. All patients with SCA occlusion underwent follow-up brain CT angiography before discharge. CT angiography source images, thick-slab maximum-intensity projection images in axial, coronal, and oblique coronal planes, and volume-rendering images were reviewed to evaluate the patency of SCAs. At CT angiography, we defined the late spontaneous recanalization as clear visualization of the ostium and whole arterial segments of previously occluded SCA. Posttreatment CT scans were evaluated to identify intracranial hemorrhages according to the Heidelberg bleeding classification^9^. Symptomatic cerebellar hemorrhage was defined, according to the Heidelberg classification, as any cerebellar hemorrhage associated with clinical evidence of neurological worsening, with the hemorrhage judged to be the principal cause of neurologic decline^9^. Malignant cerebellar infarction was defined as a cerebellar infarction causing a mass effect in the posterior cranial fossa resulting in decompressive craniectomy or in-hospital mortality^10^. All imaging examinations including DWI, angiography, CT, and CT angiography were retrospectively assessed by two neuroradiologists who were blinded to clinical information. Conclusions were made by consensus in case of disagreement between two readers.

### Clinical outcomes

Clinical outcomes were assessed by stroke neurologists using an mRS score during an outpatient visit or by telephone interviews at 90 days after endovascular therapy. A favorable outcome was defined as an mRS score of 0 to 3. We also assessed the length of hospital stay and occurrence of malignant cerebellar infarction and in-hospital mortality. Patients with in-hospital mortality were excluded from the analysis of the length of hospital stay.

### Statistical analysis

Continuous data are presented as the median and interquartile range. Categorical data are presented as the number and percentage. Comparative analysis between patients with remaining SCA occlusion and those without it was not performed in our study because the statistical power is low due to small sample size.

## Results

This study included 116 patients (63 males; median age, 73 years) who underwent DWI before and after endovascular thrombectomy for BAO. Ten patients (8.6%) had a remaining SCA occlusion on final angiograms. Eight patients had unilateral SCA occlusion and 2 patients had bilateral occlusion. Among patients with remaining SCA occlusion, the site of BAO was the distal segment in 8 patients and the middle segment in 2 patients. The baseline demographics and procedural characteristics are shown in Table 1.

**Table 1:** Baseline and procedural characteristics of the study populations.

	All patients (n = 116)	With SCA occlusion (n = 10)	Without SCA occlusion (n = 106)
Age, y	73 (63.75–80)	72 (59–76.5)	73 (64–80)
Sex, male	63 (54.3)	5 (50.0)	58 (54.7)
Vascular risk factor			
Hypertension	80 (69.0)	7 (70.0)	73 (68.9)
Diabetes mellitus	35 (30.2)	3 (30.0)	32 (30.2)
Dyslipidemia	28 (24.1)	3 (30.0)	25 (23.6)
Smoking	26 (22.4)	2 (20.0)	24 (22.6)
Atrial fibrillation	57 (49.1)	4 (40.0)	53 (50.0)
Coronary artery disease	11 (9.5)	1 (10.0)	10 (9.4)
History of stroke or TIA	19 (16.4)	1 (10.0)	18 (17.0)
Baseline NIHSS	12 (7–18.75)	16 (13.25–19)	11 (7–18)
Admission hyperglycemia^1^	51 (43.9)	4 (40)	47 (44.3)
Intravenous thrombolysis	27 (23.3)	2 (20.0)	25 (23.6)
pc-ASPECTS on DWI	7 (6–8)	6 (5.25–7)	7 (6–8)
Occlusion sites			
Proximal	25 (21.6)	0 (0)	25 (23.6)
Middle	22 (19.0)	2 (20.0)	20 (18.9)
Distal	69 (59.5)	8 (80.0)	61 (57.5)
Underlying severe ICAS	32 (24.3)	0	32 (26.7)
Stroke etiology			
Cardioembolic	56 (48.3)	5 (50.0)	51 (48.1)
Large artery atherosclerosis	43 (37.1)	2 (20.0)	41 (38.7)
Undetermined	17 (14.7)	3 (30.0)	14 (13.2)
Time to procedure	277.5 (191.25–380)	302 (202.75–477.5)	277.5 (191.25–363)
Procedure time	29.5 (18.25–46)	27 (14.5–50.75)	29.5 (19–46)
Time to reperfusion	306 (221.25–437.5)	313 (222.25–554)	306 (225.25–411)

Values are presented as n (%) or median (IQR). ^1^Admission hyperglycemia was defined as admission serum glucose of > 140 mg/dL.

*DWI* diffusion-weighted imaging, *ICAS* intracranial atherosclerotic stenosis, *NIHSS* National Institute Health Stroke Scale, *pc-ASPECTS* posterior circulation Alberta Stroke Program Early CT Score, *SCA* superior cerebellar artery, *TIA* transient ischemic attack.

**Figure 1:**
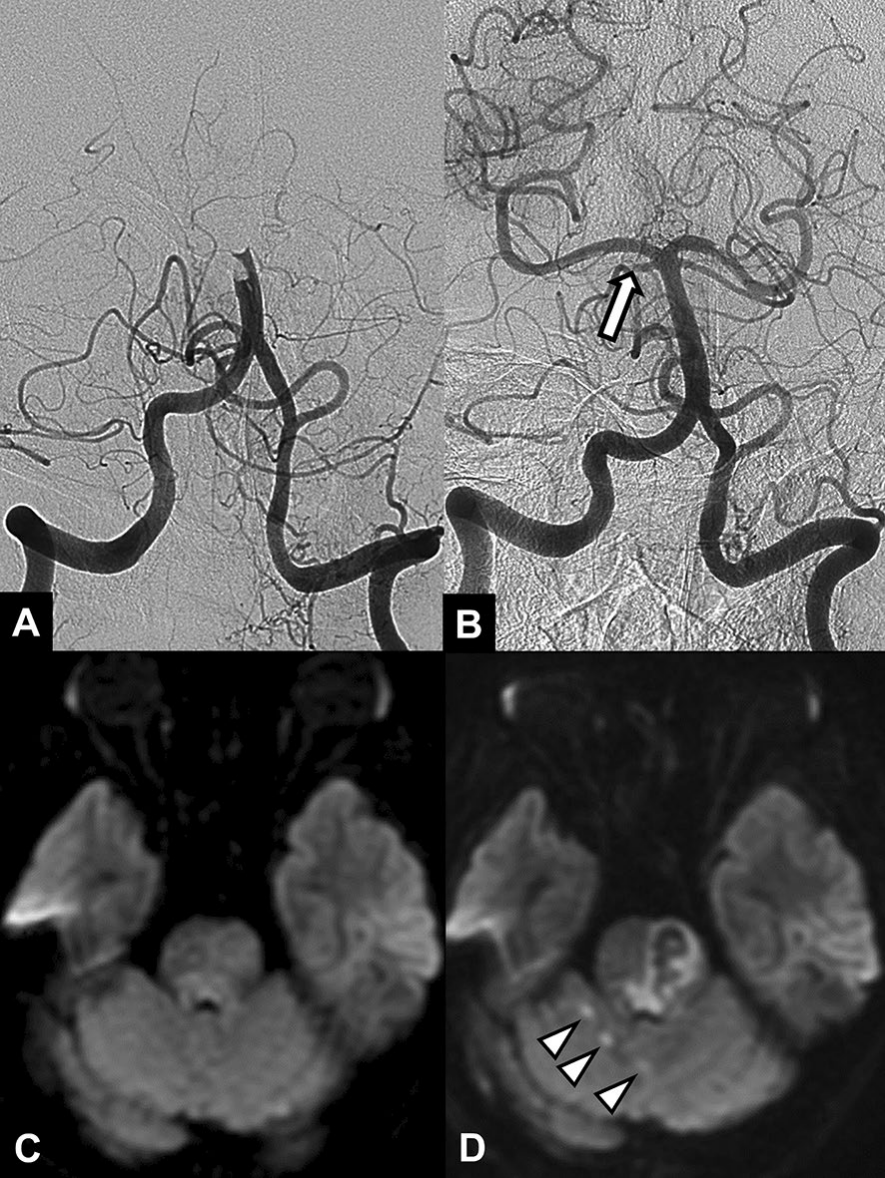
A 64-year-old female patient with acute stroke due to basilar artery occlusion. (**A**) Initial angiogram shows an occlusion at the distal segment of the basilar artery. (**B**) Final angiogram obtained after stent-retriever thrombectomy shows recanalization of the basilar artery and an occlusion (arrow) in the proximal segment of the right superior cerebellar artery. Pretreatment diffusion-weighted image (**C**) and follow-up diffusion-weighted image (**D**) show new punctate infarctions (arrowheads) in the right superior cerebellar arterial territory. Note the brain stem infarction with hemorrhagic transformation in the left half of the upper pons.

Of 10 patients with remaining SCA occlusions, 5 patients developed a new infarction in the corresponding SCA territory on follow-up DWI. (Fig. 1). All of these infarctions were punctate lesions, 1–3 in number, and 4–11 mm in diameter. Three patients with SCA occlusion showed increased extent of pre-existing SCA territorial infarction and mild mass effect on the fourth ventricle. No cerebellar infarction occurred in the remaining 2 patients. A new midbrain infarction occurred in only one patient, in the posterior region of the midbrain; this patient also had preexisting cerebellar infarction.

**Figure 2:**
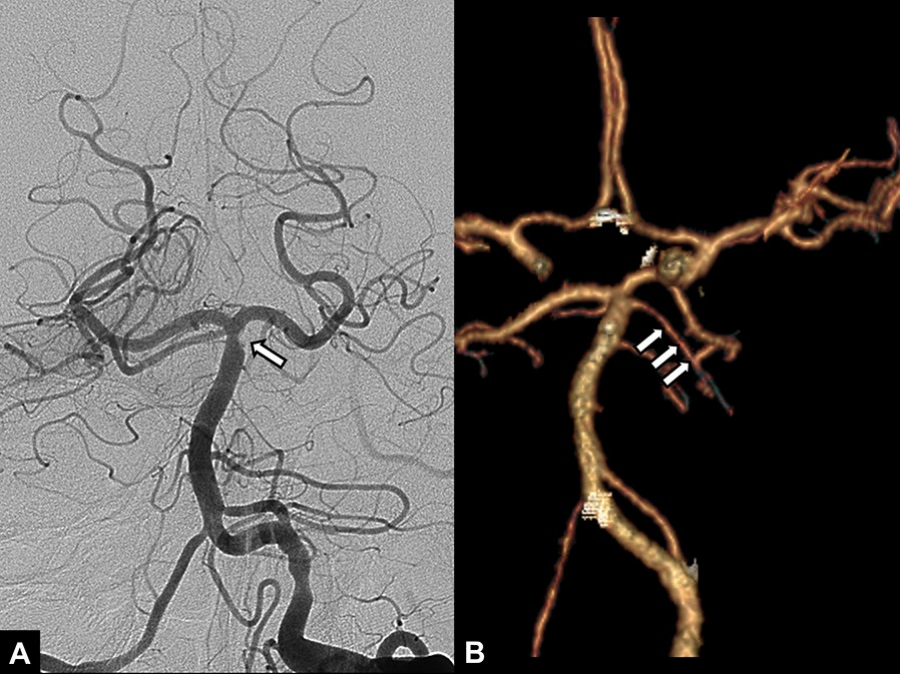
A 50-year-old male patient with acute stroke due to basilar artery occlusion. (**A**) Final angiogram obtained after stent-retriever thrombectomy shows an occlusion (arrow) at the ostium of the left superior cerebellar artery. (**B**) Follow-up CT angiography obtained 40 h after thrombectomy shows spontaneous recanalization of the previously occluded left superior cerebellar artery (arrows).

Treatment outcomes after endovascular therapy in 116 patients with acute BAO are presented in Table 2. Overall, successful reperfusion was achieved in 99.1% (115/116) of patients. The median length of hospital stay was 12 days (9–22). A 90-day favorable outcome (mRS 0–3) occurred in 50% (58/116) of patients and the rate of 90-day mortality was 10.3% (12/116). Malignant cerebellar infarction occurred in only one patient, who did not have SCA occlusion. No patients showed symptomatic cerebellar hemorrhage. The median length of hospital stay was 17 in patients with remaining SCA occlusions. Four of 10 patients with remaining SCA occlusion showed 90-day favorable outcome (mRS 0–3) and 90-day mortality occurred in one patient. At follow-up CT angiography, 9 of 12 occluded SCAs showed late spontaneous recanalization (Fig. 2).

**Table 2:** Outcomes according to the presence or absence of superior cerebellar artery occlusion.

	All patients (n = 116)	With SCA occlusion (n = 10)	Without SCA occlusion (n = 106)
Successful reperfusion	115 (99.1)	9 (90.0)	106 (100)
Symptomatic cerebellar hemorrhage	0	0	0
Malignant cerebellar infarction	1 (0.9)	0	1 (0.9%)
Length of hospital stay, days	12 (9–22)	17 (11.25–27.75)	12 (8.5–20.5)
90-day favorable outcome	58 (50)	4 (40.0)	54 (50.9)
90-day mortality	12 (10.3)	1 (10)	11 (10.4)

Values are presented as n (%) or median (IQR).

*SCA* superior cerebellar artery.

## Discussion

The SCA is the most consistent artery among the infratentorial arteries, and agenesis of the SCA either on one side or both sides has not been reported in the literature^5,6^. The orifice of the SCA can be compromised in patients with BAO, especially when clots lodge in the distal segment of the basilar artery. However, the incidence of remaining occlusion of the SCA in patients undergoing thrombectomy for acute BAO has not yet been reported. In the present study, SCA occlusion occurred in 8.6% (10/116) of such patients and the most (8 of 10) of remaining SCA occlusion occurred in patients with BAO in the distal segment.

Treatment strategies for SCA occlusion remaining after successful recanalization of the basilar artery have not yet been reported and need to be elucidated. Attempting endovascular recanalization of the occluded SCA could be one option. However, the risk of vessel injury would be high during the endovascular procedure because of the blind navigation of small arteries and acute-angled take-off of the SCAs. The results of our study suggest that attempts to recanalize remaining SCA occlusions may be unnecessary in patients with acute BAO. New cerebellar infarction occurred in only half of the patients with SCA occlusion and new midbrain infarction in only one patient in the present study. Moreover, the new infarctions were small, with a diameter ranging from 4 to 11 mm. Accordingly, no patients with remaining SCA occlusions developed symptomatic cerebellar hemorrhage or malignant cerebellar infarction in the present study. The rates of favorable outcome and mortality at 90 days in patients with remaining SCA occlusion were comparable to the results of recent randomized trials^1,2^. Therefore, this study suggests that SCA occlusions remaining after endovascular thrombectomy for BAO may have a benign prognosis.

The benign prognosis of SCA occlusions remaining after thrombectomy for BAO may be attributed to several possible mechanisms. In our study, 75% (9 of 12) of SCA occlusions seen on final angiograms had recanalized spontaneously on follow-up CT angiography. Successful recanalization of the basilar artery trunk may increase regional blood pressure on the SCA ostium and facilitate endogenous thrombolysis, both of which lead to late spontaneous recanalization. Millán et al. reported that spontaneous complete recanalization (evaluated with CT or MR angiography 24 h after stroke) occurred in 22.3% of patients with anterior circulation large vessel occlusion who received best medical management only^11^. In their study, patients with spontaneous complete recanalization at 24 h showed better clinical outcome compared with those with no or partial recanalization (90-day mRS 0–2, 57.1% vs. 23.3%). Another possible mechanism is that the recanalization of the basilar artery enhances collateral flow from the inferior cerebellar arteries and posterior cerebral artery to the SCA territory via leptomeningeal anastomoses, possibly preventing the development of large territorial infarction^12^. In addition, there may exist extensive superficial collateral anastomosis between posterior inferior cerebellar artery and SCA in some patients with BAO and this collateral might prevent severe infarction in SCA territory^13,14^.

The main limitations of this study are the small sample size and the retrospective study design. Additionally, the patency of other cerebellar arteries was not assessed in this study. However, precise assessment of the patency of inferior cerebellar arteries is difficult in patients with BAO because of anatomical variations of inferior cerebellar arteries. A CT angiographic study showed that the incidence of congenital absence of the anterior inferior cerebellar artery and posterior inferior cerebellar artery was 36.1% and 38.1%, respectively^5^. Moreover, posteroinferior cerebellar arteries are not usually occluded in patients with BAO because posteroinferior cerebellar arteries arise from the vertebral artery. Although NIHSS is the most widely used scale for assessing patients with acute ischemic stroke, it is known that NIHSS underestimates the clinical severity in posterior circulation stroke^15,16^. Several modified NIHSS systems specifically designed for posterior circulation stroke have been proposed, however, these systems require further validation in large prospective studies^16^. Similarly, mTICI system is not accurate for use in patients with vertebrobasilar artery occlusion and a specific reperfusion grading system for posterior circulation stroke need to be developed^17^. Finally, we used DWI pc-ASPECTS to assess pretreatment extent of infarction in this study. It is well known that there is a general shift towards lower score with DWI compared to CT angiography source image or noncontrast CT scan.

In conclusion, our results showed that SCA occlusions remained in 8.6% of patients undergoing endovascular thrombectomy for acute BAO, but they had a benign clinical course. In addition, most SCA occlusions had recanalized spontaneously on follow-up CT angiography. These results suggest that attempts to recanalize remaining SCA occlusions may be unnecessary in patients with acute BAO.

## Data Availability

The data that support the findings of this study are available from the corresponding author, upon reasonable request.
